# Sexual dimorphisms in the transcriptomes of murine salivary glands

**DOI:** 10.1002/2211-5463.12625

**Published:** 2019-03-30

**Authors:** Taro Mukaibo, Xin Gao, Ning‐Yan Yang, Maria S. Oei, Tetsuji Nakamoto, James E. Melvin

**Affiliations:** ^1^ Secretory Mechanisms and Dysfunctions Section National Institute of Dental and Craniofacial Research National Institutes of Health Bethesda MD USA; ^2^ Division of Oral Reconstruction and Rehabilitation Kyushu Dental University Kitakyushu, Fukuoka Japan; ^3^ Joint Institute for Food Safety and Applied Nutrition University of Maryland College Park MD USA; ^4^ Department of Pediatric Dentistry Beijing Stomatological Hospital & School of Stomatology Capital Medical University Beijing China; ^5^ Department of Prosthodontics Matsumoto Dental University Shiojiri Japan; ^6^ Present address: School of Dentistry University of Maryland College Park MD USA

**Keywords:** Cftr, ENaC, NaCl reabsorption, RNA‐seq, salivary gland, sexual dimorphism

## Abstract

Transcriptional profiling identified 933 sexually dimorphic genes out of the 14 371 protein‐coding genes expressed in the three major murine salivary glands: parotid, sublingual, and submandibular. Most (89%) sex‐specific genes were enriched in a single gland, while only 0.5% of the sexually dimorphic genes were enriched in all glands. The sublingual gland displayed a strong male sex bias (94% of sex‐enriched genes), while a sex preference was not obvious in the parotid or submandibular glands. A subset of transcription factor genes was correlated with the expression of gland‐specific, sex‐enriched genes. Higher expression of Cftr chloride and Scnn1 sodium channels in the male submandibular correlated with greater NaCl reabsorption. In conclusion, adult salivary glands display sex‐ and gland‐specific differences in gene expression that reflect their unique functional properties.

**Abbreviations**
FPKM: fragments per kilobase of transcript per million mapped reads
GO: Gene Ontology
PCA: principal component analysis
PG: parotid gland
qPCR: quantitative PCR
SEG: sex‐enriched gene
SLG: sublingual gland
SMG: submandibular gland
TF: transcription factor

Sexual dimorphism refers to male and female differences in metabolic, physiological, and morphological traits observed in many organs such as brain anatomy and function 1, 2, muscle physiology 3, 4, and salivary gland morphology 5. Sexual dimorphism of salivary glands was originally noted more than 50 years ago and has been most frequently studied in the murine submandibular gland (SMG) where differences in gland structure 6 and for the expression of biologically active proteins such as nerve and epithelial growth factors 7, 8 have been reported. Sex‐related differences in gene expression have been described in mammalian salivary glands by microarray with a limited number of gene probes and analysis strategies 9, 10, 11, while mouse SMG development was previously assessed by RNA sequencing (RNA‐seq) transcriptomic analysis but sex was not ascertained for embryonic and early postnatal tissues and only male tissues were included for older time points 12. Consequently, a comprehensive deep sequencing approach has not been previously used to investigate sexual dimorphism of the three major mammalian salivary glands.

Salivary gland parenchyma is composed of acinar and ductal cells, although there are unique differences in these two general cell types in the parotid (PG), SMG, and sublingual glands (SLG). Parotid and sublingual acinar cells are largely serous and mucous, respectively, while rodent submandibular acinar cells are termed seromucous. The intralobular ducts of the parotid and sublingual are primarily composed of striated cells, but the ducts of the rodent submandibular also contain a large population of sexually dimorphic granular convoluted tubule cells, expansion of which is associated with puberty 13. As originally described 14, saliva is generated in two stages. Acini produce a primary saliva that is plasma‐like, NaCl‐rich fluid, which is subsequently modified when much of the NaCl is reabsorbed during passage through the ducts 15, 16. Mice deficient in expression of individual ion transport proteins have provided important mechanistic information about acinar fluid secretion and ductal NaCl reabsorption processes, but given the limitations of genetic approaches, only a relatively few number of candidate genes have been investigated and it is not clear if these observations universally apply to all major salivary glands. Such studies have paid little attention to sex‐specific gene expression patterns related to salivary gland function.

The molecular and functional consequences of sex‐specific differences in gene expression are largely unexplored in the major mammalian salivary glands. Thus, the aim of the current study was to identify the sex‐specific differences in the transcriptional profiles of the three major salivary glands that may have functional consequences. We found striking differences in the transcriptomes of male and female glands that associated with unique gene expression programs in each major gland type. Sublingual glands displayed a very strong male bias for sex‐enriched genes (SEGs), while the SMG expressed the greatest number of sexually dimorphic genes, including the *Cftr* (cystic fibrosis transmembrane conductance regulator) and *Scnn1* (sodium channel epithelial) ion channel genes, differential expression of which directly correlated with a sex‐specific difference in NaCl reabsorption in SMG.

## Materials and methods

### General methods

Experiments were performed using 9‐ to 16‐week‐old adult mice housed in pathogen‐free, micro‐isolator cages with free access to laboratory chow and water with a 12‐h light/dark cycle. Animals received humane care in compliance with the Guide for the Care and Use of Laboratory Animals published by the National Institutes of Health, USA. Animal procedures were approved by the Animal Care and Use Committee of the National Institute of Dental and Craniofacial Research, National Institutes of Health (ASP 16‐802). Chemicals were purchased from Sigma‐Aldrich (St Louis, MO, USA) unless otherwise stated.

### RNA sequence data and analyses

RNA sequencing libraries were generated for the PG, SMG, and SLG from three male and three female adult *C57BL/6J* mice (Jackson Laboratories) as previously described 17. The data were subjected to analysis using the platform provided by DNAnexus (Mountain View, CA, USA) and then:

- Mapped against the mouse mm10 reference genome downloaded from Illumina iGenomes: https://www.ncbi.nlm.nih.gov/geo/query/acc.cgi?token=mrwlyaoqpjmfpir&acc=GSE96747 with STAR 2.4.0j,
- Measured for expression levels of genes and transcripts with Cufflinks 2.2.1, and
- Compared across the three major salivary glands and sexes with Cufflinks/Cuffdiff 2.2.1 for significance (*q *<* *0.05).

Gene expression level was expressed as fragments per kilobase of transcript per million of mapped reads (FPKM), which were scaled via the median of the geometric means of fragment counts across all sequencing libraries. Sex‐specific differentially expressed genes were identified from each gland with a *q*‐value of 0.05 as cutoff. Briefly, total RNA was extracted from the 18 salivary glands and cDNA synthesized (Otogenetics Corporation, Atlanta, GA, USA). Illumina libraries were made from fragmented cDNA and submitted for Illumina HiSeq 2500 sequencing. Each of the 18 sample libraries were sequenced at ~ 40 million reads, with sufficient depth to define differences in gene expression. The principal component analysis (PCA) plots, heat maps, and cluster dendrograms created in R were self‐organizing for both samples and genes using the FPKM values from the 18 individual samples. Gene Ontology (GO) term annotations were derived from the GO Consortium database (http://www.geneontology.org), which was further supplemented with InterProScan output 18. The GO enrichment analysis was performed using func packages 19.

### *In vivo* saliva collection and analyses

*C57BL/6J* (Jackson Laboratories) and *BlackSwiss‐129X1/SvJ* hybrid (in‐house colony originally obtained from the University of Cincinnati) mice were rendered unconscious by intraperitoneal injection of chloral hydrate (400 mg·kg^−1^ body weight) and *in vivo* saliva secretion stimulated by intraperitoneal injection of pilocarpine/HCl (10 mg·kg^−1^ body weight). Briefly, gland‐specific saliva was separately collected for 30 min from the PG, SMG, and SLGs by insertion of the cut ends of the ducts into individual capillary tubes. Saliva samples were stored at −86 °C. The sodium concentration was analyzed by atomic absorption spectroscopy (PerkinElmer Life Sciences 3030 spectrophotometer, Waltham, MA, USA), and the chloride activity was measured using a digital expandable ion analyzer (EA940; Orion Research, Beverly, MA, USA).

The magnitude of NaCl reabsorption by each gland was estimated as previously described 20. Briefly, the measured [NaCl] values were subtracted from the [NaCl] reported for the primary saliva collected by micropuncture for mouse salivary glands 21. The [NaCl] in the primary saliva represents the NaCl concentration of the acinar lumen, and thus, subtraction of the final saliva [NaCl] as it exits the main duct from the primary saliva [NaCl] estimates the amount of NaCl reabsorption by the salivary gland ducts. NaCl reabsorption was expressed as mEq, considering the volume of saliva secreted by each gland, and compared in Fig. 5 to the average flow rate during a 30‐min collection period. Differences were called statistically significant (*P *<* *0.05) as determined using an unpaired Student's *t*‐test (Origin 2017, OriginLab Corp, Northampton, MA, USA).

### Quantitative PCR analyses

Quantitative PCR (qPCR) was performed as previously described 22. Briefly, SMG, PG, and SLG from four female and four male *C57BL/6* mice were immediately frozen in liquid nitrogen and shipped to MyOmicsDx (Towson, MD, USA) for processing. Total RNA was isolated and reverse‐transcribed to synthesize cDNA. Real‐time qPCRs in four replicates were performed to determine the mRNA levels for members of the Na^+^ channel *Scnn1* gene family (*a, b*, and *g*) and the Cl^−^ channel *Cftr* using Bio‐Rad CFX96 Touch™ Real‐Time PCR Detection System (Bio‐Rad, Hercules, CA, USA) with specific primer pairs. The β*‐actin* mRNA level was selected as reference for standard −ΔΔCq normalization. Expression correlation across the salivary gland samples was calculated for individual genes between the qPCR abundance values and RNA‐seq FPKM values.

## Results

### Sex‐specific differences in the transcriptomes of murine salivary glands

**Figure 1:**
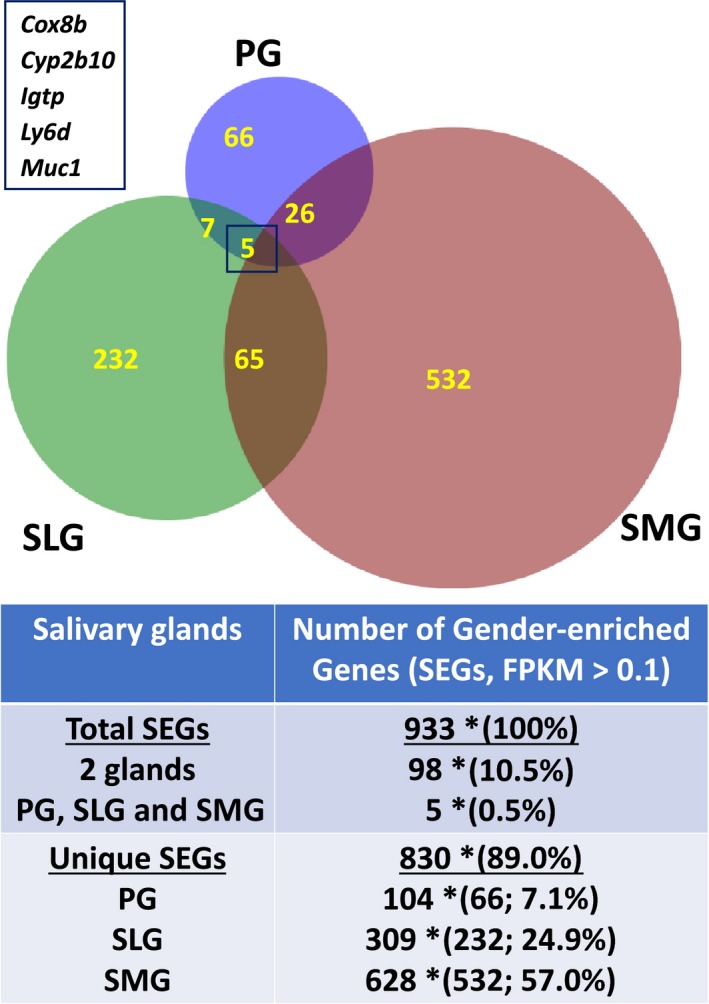
Comparison of SEGs among the murine major salivary glands. Venn diagram: the relationship between the number SEGs in the PG, SLG, and SMG, as listed in Table S1, with an FPKM cutoff value of ≥ 0.1, *q *< 0.05. Inset: list of five common SEGs in all three salivary glands. Table: total SEGs: total number of SEGs expressed in the three salivary glands (*n* = 933), number of SEGs expressed in at least two salivary glands (*n* = 98), and number of SEGs found in all three salivary glands (*n* = 5). *Data in brackets represent the percentage of SEGs in two (10.5%) or all three gland types (0.5%) relative to the total number of expressed SEGs (100%). Unique SEGs: total number of uniquely expressed SEGs in the three salivary glands (*n* = 830, 89% of total expressed SEGs) and the total number of SEGs found in individual salivary glands (PG = 104, SLG = 309, SMG = 628). *Data in brackets represent the number and percentage of unique SEGs in a single gland type relative to the total number of SEGs expressed in all three salivary glands. *n* = 6 for each gland type consisting of three male and three female glands.

RNA sequencing data acquired from the three major salivary glands of adult male and female *C57BL/6J* mice were described previously and the full data sets deposited in the Gene Expression Omnibus (GSE96747) 17. Sex‐specific analysis of the mouse salivary gland transcriptomes revealed that of the 14,371 protein‐coding genes detected (FPKM cutoff ≥ 0.1), 6.5% were sex‐enriched at significantly different levels (933 out of 14 371 genes, *q *<* *0.05) in the PG, SMG, and SLG of adult male and female mice (Table S1). The PG transcriptome displayed the least number of sex‐specific genes (*n* = 104 genes), followed by the SLG and SMG (*n* = 309 and 628 genes, respectively; Fig. 1).

It was striking that 89% of the SEGs (*n* = 830 out of 933) were differentially expressed in a single gland type (Fig. 1: gland‐specific, SEGs, PG = 66; SLG = 232; SMG = 532), demonstrating that sex‐specific expression programs are overwhelmingly enriched in a single gland. In contrast, 10.5% of the SEGs were differentially expressed in two out of the three glands (*n* = 98), while expression of only five sex‐specific genes overlapped in all three glands (0.5% of SEGs). The functional interrelationship of these five common SEGs (Fig. 1, inset) is unclear, but they include *Cox8b* (cytochrome c oxidase subunit VIIIb), *Cyp2b10* (cytochrome P450, family 2, subfamily b, polypeptide 10), *Igtp* (interferon‐gamma‐induced GTPase), *Ly6d* (lymphocyte antigen 6 complex, locus D), and *Muc1* (mucin 1).

**Figure 2:**
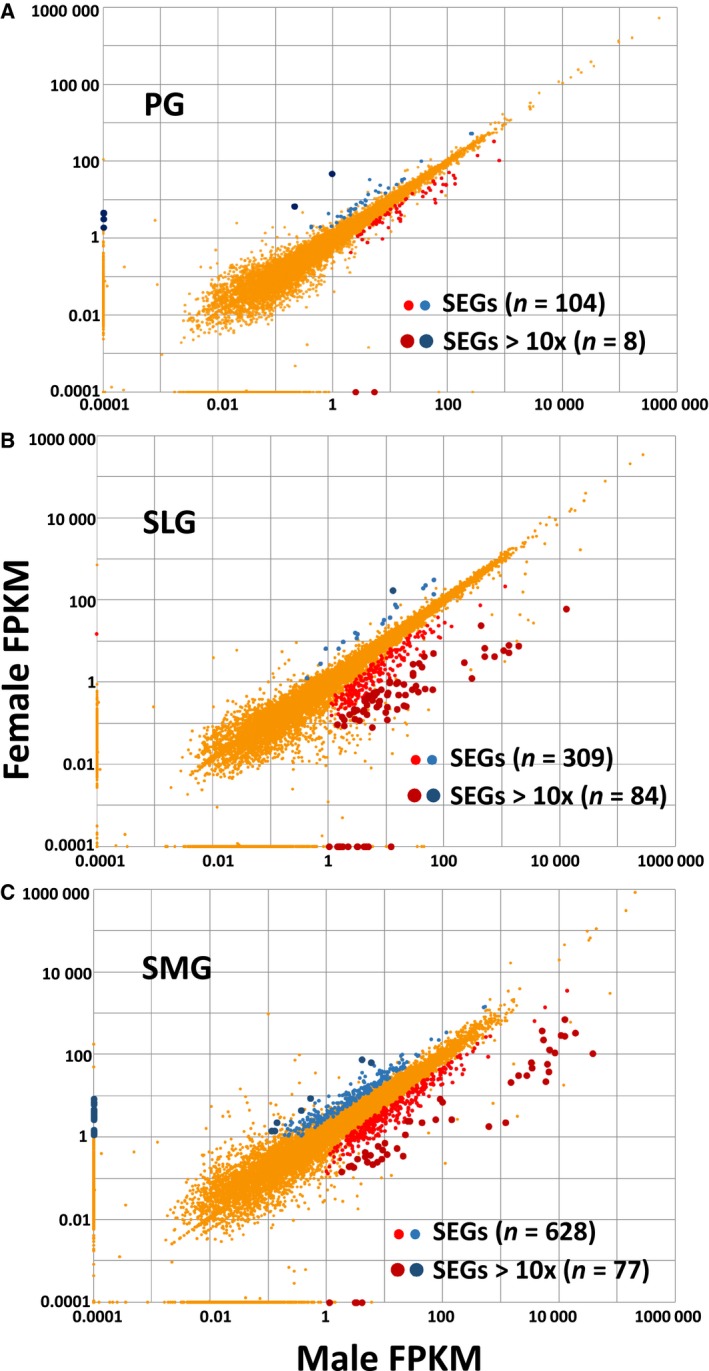
2D‐FPKM scatterplot of sex‐wise differential gene expression in murine PG, SLG, and SMG. Gene expression levels expressed as FPKM values of male vs. female transcriptomes (blue and red circles represent female‐ and male‐enriched genes, respectively) are plotted for the PG, SLG, and SMG. (A) The 104 differentially expressed, SEGs in the PG displayed no obvious sex bias (*n* = 61 > male SEGs out of 104, 59%), with eight SEGs expressed at 10× greater levels (large circles). (B) Of the 309 SEGs in the SLG, 290 (94%) were more highly expressed in males, with 85 SEGs expressed at 10× greater levels (large circles). (C) The SEGs in the SMG displayed no strong sex preference (*n* = 354 out of 628 > males, 56%), while 77 of the SEGs were expressed at > 10× (larger dark circles).

2D‐FPKM scatterplots of male vs. female gene expression levels (red vs. blue circles, respectively) for the PG, SMG, and SLG are shown in Fig. 2. Nearly all the 309 SEGs in the SLG were more highly expressed in males (*n* = 290, 94%; Fig. 2B), and 85 of these SEGs (84 in the male SLG) were more highly expressed at 10× greater levels (large circles). Eleven of the male SLG SEGs had FPKM values > 100×, which included nine members of the *Klk* gene family (kallikreins) and nerve growth factor (*Ngf*). In contrast to the SLG, the SEGs displayed no obvious sex bias in the PG (59% > males, *n* = 61 out of 104 SEGs; Fig. 2A) or in the SMG (56% > males, *n* = 354 out of 628 SEGs; Fig. 2C). The SMG SEGs with a > 10× sex difference showed a relatively weak male bias (65%, *n* = 49 out of 77), and 18 of these latter genes were male dominant with FPKM values > 1000×, including 12 members of the *Klk* gene family as well as *Ngf*,* Amy1* (amylase 1), *Cst10* (cystatin 10), *Dccp1* (demilune cell and parotid protein 1), and *Bpifa2* (BPI fold‐containing family A, member 2; Psp). Many of these latter known secretory proteins are specifically expressed in acinar (e.g., amylase and Dccp1) or duct cells (e.g., NGF).

**Figure 3:**
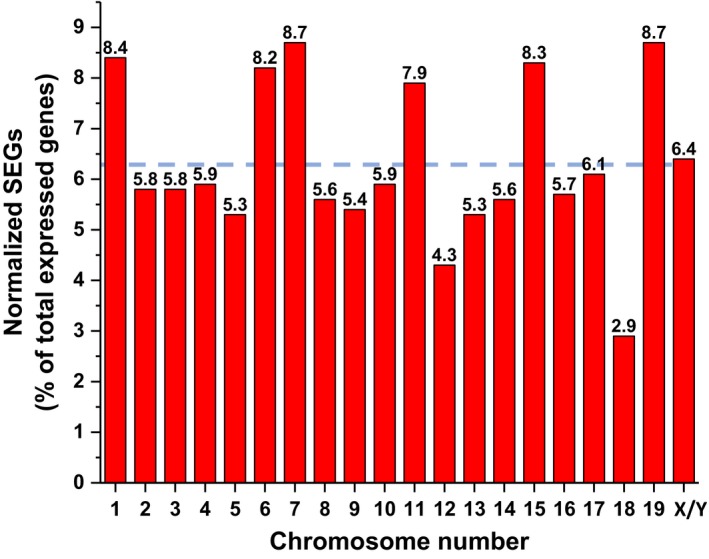
Percentage of SEGs by chromosome number in the murine major salivary glands. Sexually dimorphic genes randomly distributed across the 20 mouse chromosomes. The percentage of SEGs (FPKM ≥ 0.1 cutoff) was normalized to the total number of expressed protein‐coding genes on a given chromosome (see ref. 17). The percentage is given above each bar for the corresponding chromosome. The dashed blue line represents the average number of SEGs for the 20 mouse chromosomes (mean = 6.3%).

A limited number of the 933 sexually dimorphic genes were located on the X and Y chromosomes (*n* = 30, 3.2%), demonstrating that most of the SEGs in salivary glands are autosomal genes common to both sexes (> 96%; Table S1). Furthermore, ~ 6.3% (range: 2.9–8.7%) of the protein‐coding genes on a given chromosome were SEGs when normalized to the total number of expressed genes per chromosome (Fig. 3), demonstrating that the sexually dimorphic genes are randomly distributed across the 20 mouse chromosomes.

A total of 289 enriched GO terms were associated with the SEGs (*P *<* *0.005), including 190 ‘biological process’ terms, 42 ‘cellular component’ terms, and 57 ‘molecular function’ terms (Table S2). Because most of the GO terms (92%, 265 out of 289) were unique to only one salivary gland, we posited that each gland expresses a unique functional spectrum of SEGs. In contrast, four of the 289 GO terms were common to all three glands (1.4%, four out of 289 GO terms) and these terms were related to either ‘immune responses’ (GO:0035458—response to interferon‐beta; GO:0071346—response to interferon‐gamma) or ‘membrane dynamics’ (GO:0009986—cell surface; GO:0070062—extracellular exosome). The functional significance of sex‐enriched expression of immune response genes in all three major salivary glands is unclear, but given that salivary glands are constantly exposed to foreign molecules and potential pathogens in the oral cavity that might elicit active immune responses, tight regulation is apparently desirable. Another major function of salivary glands is stimuli‐regulated secretion. The overall functional enrichment profile indicated that male and female salivary glands have unique responses to different types of signaling molecules, for example, *Ngfr* and *Tgfb2* (Table S2).

### Correlation between sexually dimorphic and transcription factor genes

**Figure 4:**
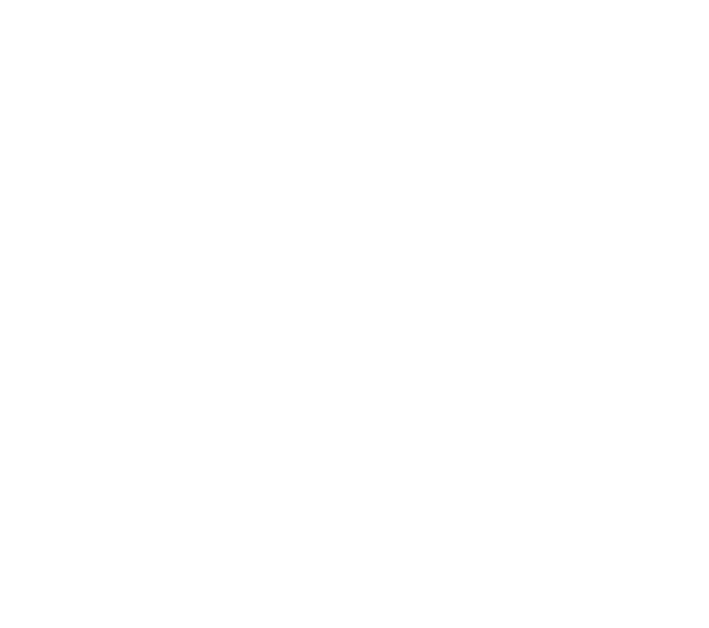
Cluster analysis of sex‐enriched vs. TF genes in the murine major salivary glands. Pairwise Pearson correlation coefficients were calculated with a cutoff of 0.9 between the SEGs (*n* = 933) and SE TFGs (*n* = 51) based on FPKM across the 18 gland samples and used to indicate the co‐expression for each pair. Of the 933 SEGs, one region was revealed where no clustering occurred, and these 262 SEGs were not considered. When the remaining 671 sex‐specific SEGs were correlated with the 51 SE TFGs, three prominent clusters were identified that included 205, 189, and 36 (430 total) sexually dimorphic genes that associated with 10 (SLG, blue box), seven (SMG, red box), and four (SMG, green box) SE TFGs, respectively (gene IDs given in Table S3; red = male SE TFGs, blue = female SE TFGs).

Of the 1675 documented mouse transcription factor (TF) genes (http://genome.gsc.riken.jp/TFdb/ 23), 51 were differently expressed by sex across the three glands. To better understand how gene co‐expression networks reflect the association between sexually dimorphic genes and their regulatory factors, we evaluated how SEGs and sex‐enriched TF genes (SE TFGs) cluster based on co‐expression patterns (Fig. 4). Pairwise Pearson correlation coefficients between the SEGs (*n* = 933) and SE TFGs (*n* = 51) across the 18 gland samples were calculated with a cutoff of 0.9 for the Pearson correlation coefficient to indicate the co‐expression for each pair.

We found that 262 of the 933 SEGs did not correlate with expression for any of the 51 sex‐enriched TFGs, and therefore, they were not further considered for analysis. When the remaining 671 SEGs were correlated with expression of the 51 SE TFGs, three prominent clusters were identified (Fig. 4) that included 205, 36, and 189 sexually dimorphic genes that associated with 21 of the 51 SE TFGs in a gland‐specific pattern, that is, 10 (*SLG*, blue box), seven (*SMG*, red box), and four (*SMG*, green box) differentially expressed SE TFGs, respectively (gene IDs given in Table S3). Of note, none of the differentially expressed SEGs in the PG correlated with SE TFGs, consistent with the PG displaying the least sexual dimorphism of the three major salivary glands. All 10 SLG‐specific SE TFGs (blue cluster) were more highly expressed in males. Given that 94% of the differentially expressed genes in the SLG (290 out of 309 genes) were more highly expressed in males (see Fig. 2), one might speculate that these 10 male‐enriched TF genes are positive regulators for expression of the male‐enriched genes in the SLG. In contrast, the seven SMG‐specific red cluster GE TF genes were more highly expressed in females while the four SMG‐specific green cluster GE TF genes were more highly expressed in males, again, consistent with little, gender bias in the SMG.

Functional enrichment analysis of the SEGs whose expression correlated with SE TFGs showed that these three clusters (Table S4) generally did not overlap with the GO terms associated with the sex‐wise differentially expressed genes (Table S2). Of note, however, the genes in the SLG:blue cluster (10 SE TFGs and 205 SEGs) associated with GO terms involved in immune responses/signal transduction, including three of the above common GO terms (GO:0035458, GO:0071346, and GO:0070062). The SMG:green cluster (four DE TFGs and 189 DEGs) associated with secretion functions, while the SMG:red cluster (seven DE TFGs and 36 DEGs) associated with transcriptional regulation terms.

### Sex‐specific differences in mRNA expression of ion transport‐related genes

**Figure 5:**
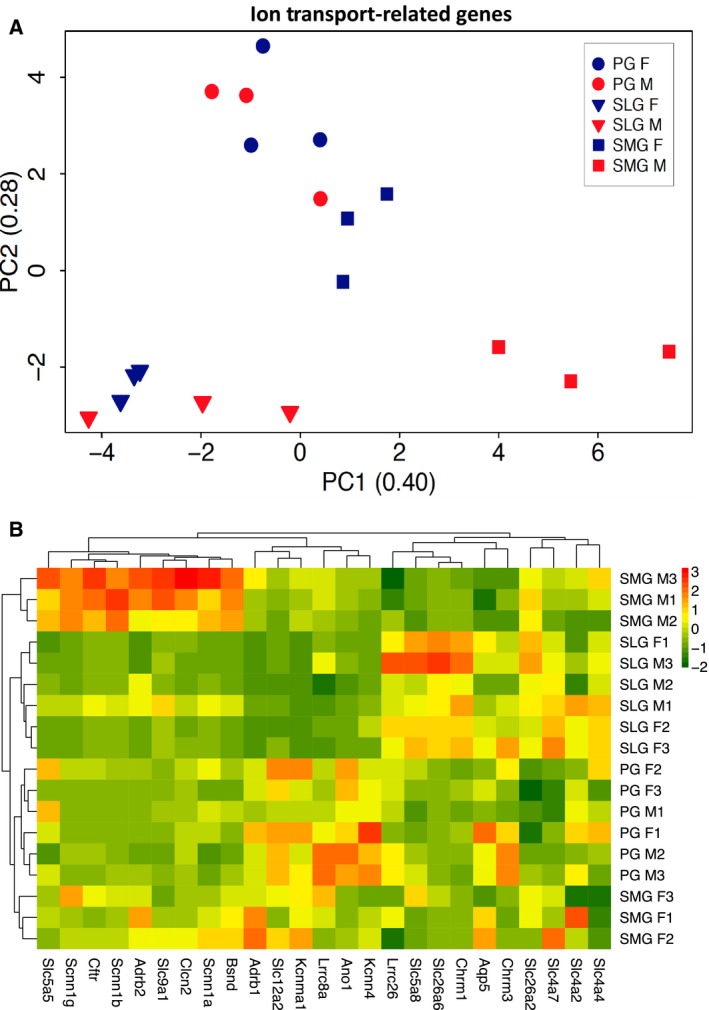
Comparison of the major ion transport‐related genes expressed in murine PG, SLG, and SMG. (A) PCA plot compares the transcriptional profiles of 25 ion transport‐related genes obtained by RNA‐seq of female (blue symbols) and male (red symbols) mice from PG (circles) in comparison with SLG (triangles) and SMG (squares) glands. A plot of the first two PC1–2 accounted for 68% of the data variation. The data segregated into three major clusters by gland type and/or sex: cluster 1—male and female SLG (blue and red triangles, *n* = 6); cluster 2—male and female PG (blue and red circles, *n* = 6) with female SMG (blue squares, *n* = 3); and cluster 3—male SMG (red squares, *n* = 3). *N* = 6 for each gland type and three for each sex/gland type. (B) Heat map self‐organized for the 18 gland samples and the 25 ion transport‐related genes. The 25 ion transport‐related genes used to generate the plots in panels A and B are given in Table S5.

As mentioned above, stimuli‐regulated secretion is a major function of salivary glands. To compare the fluid and electrolyte secretion mechanism of the three major mammalian salivary glands, PCA was performed on 25 ion transport‐related genes generally accepted to be physiologically important for salivary gland secretion (Fig. 5A). When the first two principal components (PC1–2) were plotted, 68% of the data variation was accounted for. The 18 samples clustered into three groups by gland type and/or sex (male and female = red and blue symbols, respectively). The PG (circles) and SLG (triangles) clustered into two distinct groups with no apparent sex preference. In contrast, the SMG segregated into two discrete subgroups where female SMG (blue squares) closely associated with the PG cluster, while the male SMG (red squares) clustered independently of the parotid, sublingual, and female submandibular. A heat map comparison also revealed that the 25 ion transport‐related genes self‐organized into three clusters by gland type and/or by sex (Fig. 5B). The male SMG cluster correlated most strongly with a group of nine highly expressed ion transport‐related genes, which included the Cl^−^ channel *Cftr*, the α, β, and γ subunits of the Na^+^ channel *Scnn1*, and the Na/H exchanger *Nhe1*.

The average gene expression for the 25 ion transport‐related genes (FPKM values) used to generate Fig. 5A,B is shown for the PG, SMG, and SLG in Table S5A (by gland) and Table S5B (by gland and sex). The expression levels were not significantly different among the three glands or between male and female mice for the aquaporin‐5 water channel/*Aqp5* and the Na^+^/K^+^/2Cl^−^ cotransporter (Nkcc1)/*Slc12a2*, suggesting that these two key fluid secretion‐related pathways in salivary acinar cells 24, 25, 26 are important in all major salivary glands regardless of sex. Of note, the Ca^2+^‐activated anion channel *Ano1* was expressed at nearly threefold higher levels in PG than SMG and SLG, consistent with the greater functional expression of Ano1 protein in the murine parotid salivary gland 27, but *Ano1* expression was not sexually dimorphic in mouse salivary glands (Table S5B). In contrast, the epithelial Na^+^ channel (ENaC) subunits/*Scnn1* and Cl^−^ channels/*Cftr*, whose coordinated activities drive NaCl reabsorption in many epithelial tissues 28, 29, 30, were more highly expressed in the SMG than the PG and SLG (Table S5A) and this was primarily due to significantly higher expression in the male SMG (Table S5B). The SMG male bias was similarly dramatic for the β subunit of the epithelial sodium channel/*Scnn1b*, and a similar pattern was observed for the α and γ subunits of *Scnn1*. The differences in the expression levels of the *Cftr* and *Scnn1* genes were verified by qPCR (Table S5C). In addition, the male SMG displayed higher expression of the voltage‐dependent anion channel ClC‐2/*Clcn2* and the sodium–proton exchanger Nhe1/*Slc9a1*.

### NaCl reabsorption by the three major murine salivary glands

**Figure 6:**
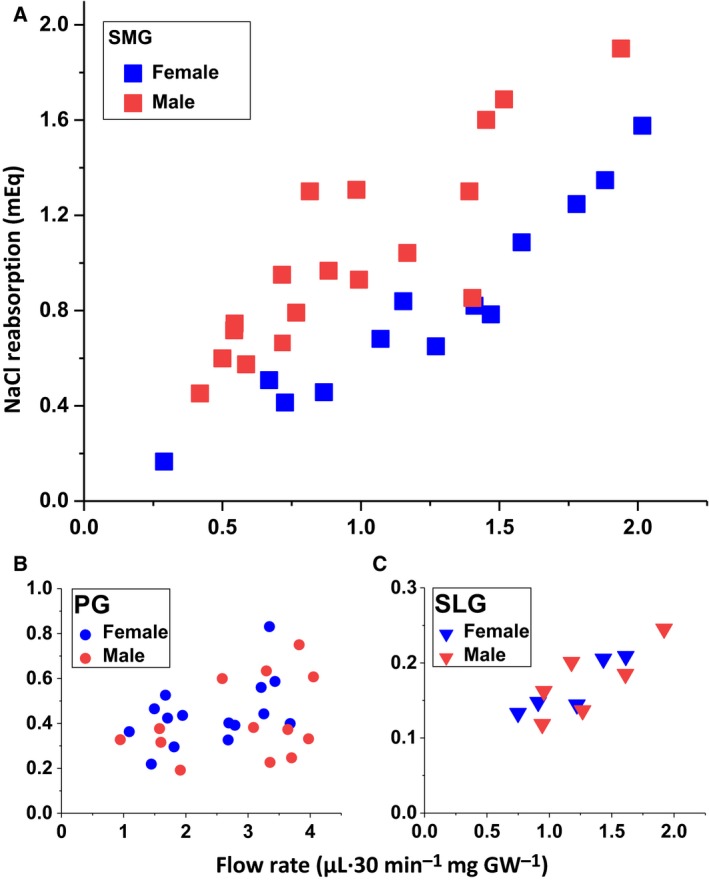
Correlation of *in vivo* stimulated flow rate with NaCl reabsorption in murine PG, SLG, and submandibular salivary glands. The SMG, PG, and SLG of female and male BlackSwiss‐129X1/SvJ mice were stimulated *in vivo* by the cholinergic agonist pilocarpine (intraperitoneal 10 mg·kg^−1^ body weight) for 30 min, and the magnitude of NaCl reabsorption in saliva was estimated (see [Link], [Link], [Link], [Link], [Link]). The magnitude of NaCl reabsorption (mEq) vs. the flow rate (μL·30 min^−1^·mg^−1^ GW, where GW, gland weight) is plotted. (A) The SMG(squares), (B) PG (circles), and (C) SLG (triangles) glands were analyzed by sex, where female SMG, PG, and SLG (blue symbols) *n* = 13, 15, and 5 glands, respectively, and male SMG, PG, and SLG (red symbols) *n* = 18, 13, and 6 glands, respectively.

Beyond the *in silico* whole‐transcriptome profiling, we also performed *in vivo* functional studies to better understand how the above gland‐ and sex‐specific differences in mRNA expression of ion transport‐related genes impact the NaCl reabsorption mechanism. Saliva was collected *in vivo* from individual glands of adult mice and the amount of NaCl reabsorption determined. The SLG of *C57BL/6J* mice secrete insufficient saliva for this purpose; consequently, saliva was also collected from *BlackSwiss‐129X1/SvJ* mice. In both mouse backgrounds, the [NaCl] of submandibular saliva (SMG—*C57BL/6J*: 60.6 ± 5.2 mm,* n* = 21; *BlackSwiss‐129X1/SvJ*: 55.6 ± 4.1 mm,* n* = 38) was severalfold less compared to parotid (PG—*C57BL/6J*: 145.5 ± 4.1 mm,* n* = 21; *BlackSwiss‐129X1/SvJ*: 178.1 ± 6.0 mm,* n* = 37). These results demonstrate that NaCl reabsorption is significantly more robust in SMG of both mouse strains, which is consistent with higher expression of *Cftr* and *Scnn1* subunits (Tables S5B,C). The *in vivo* NaCl concentration of the SLG of *BlackSwiss‐129X1/SvJ* mice was intermediate to the PG and SMG (SLG—76.4 ± 4.4 mm,* n* = 13). Figure 6A shows that the male SMG reabsorbed more NaCl (mEq of NaCl) than the female SMG at all comparable flow rates, in keeping with the higher expression levels of Na^+^ and Cl^−^ channel genes in male SMG. Of note, there was considerable overlap with no apparent difference in NaCl reabsorption at comparable flow rates by the male and female PG or SLG (Fig. 6B,C, respectively), in agreement with the lack of a sex bias for expression of Cftr and ENaC mRNAs in PG and SLG.

## Discussion

Transcriptional profiling revealed that out of the more than 14 371 protein‐coding genes expressed by the murine parotid, submandibular, and sublingual salivary glands, 933 were sexually dimorphic. The SEGs were randomly distributed across all 20 chromosomes, indicating that nearly all sexually dimorphic traits in salivary glands are associated with autosomal genes common to both sexes. Given that the three major salivary glands have comparable embryonic origins and functions, it was striking that the transcriptional profile of the sexually dimorphic genes was unique to each gland; that is, nearly 90% of the SEGs were differently expressed in only one of the major salivary glands. Consistent with this latter observation, nearly all GO terms associated with SEGs were also unique to a given salivary gland. However, one prominent difference is that GO terms related to immune responses were common among the three mouse major salivary glands. A sex difference in the expression of immune modulators was also noted for human minor salivary glands 31, suggesting that mouse salivary glands may provide a good model to better understand sex differences in immune response.

A functional consequence of salivary gland‐specific sexual dimorphism observed in the present study was that NaCl reabsorption was greater in the male submandibular. Salivary ducts are responsible for NaCl reabsorption, and these cells express at least two types of Cl^−^ channels, the hyperpolarization‐activated Clcn2 32 and cAMP‐dependent Cftr 33 Cl^−^ channels. Expression of *Clcn2* mRNA was more than threefold greater in the male SMG. However, it was previously reported that NaCl reabsorption is unaffected in *Clcn2* null mice, suggesting that this channel does not play a major role in NaCl reabsorption 34. On the other hand, transcriptomic analysis revealed that the male submandibular expressed higher mRNA levels of the chloride channel/*Cftr* and the sodium channel/*Scnn1*. Note that salivary glands rely on Cftr and Scnn1 channels to mediate NaCl reabsorption 33, a well‐recognized NaCl reabsorption mechanism in other epithelial tissues such as sweat gland, lung, and kidney 28, 29, 30.

Although the mechanism responsible for the sex bias in the expression of *Cftr* and *Scnn1* genes in the SMG is unknown, these genes are regulated by sex hormones in other epithelial tissues 35, 36, 37, 38. Moreover, the nature of the effects of sex hormones on expression of Cftr and Scnn1 ion channels appears to be organ specific in epithelial tissues. Regardless of the mechanism, the simplest explanation for the sex‐specific functional differences in male and female murine SMG, but not in the PG and SLG, is most likely related to the greater contribution of granular convoluted tubular ducts to gland mass in the male SMG. Ducts represent about 45–55% of the gland mass in the male SMG while the female gland is about 25–30% 20, 27. However, we cannot rule out other forms of sex‐specific regulation of these channels or other ion transporter mechanisms that may ultimately regulate the NaCl reabsorption process in male and female SMG as noted in rat kidney 39. Of note, disruption of Nhe1 (*Slc9a1*) expression dramatically impairs NaCl reabsorption in the mouse PG 40, suggesting that Nhe1 may also be involved in the sexual dimorphism of NaCl reabsorption.

In summary, striking differences were found in the transcriptomes of male and female salivary glands that correlated with unique gene expression programs in each gland type, including transcription factors that likely regulate and underlie the sex‐specific functional properties of salivary glands. Analysis of such information may lead to the generation of new functional models to better test and define the unique phenotypic properties of the different types of salivary glands. Given that there are currently no effective treatments for salivary gland dysfunction, a better understanding of the molecular and physiological properties of the three major salivary glands may identify sex‐specific therapeutic targets to improve oral health of males and females suffering from xerostomia.

## Conflict of interest

The authors declare no conflict of interest.

## Author contributions

JEM conceived and supervised the study; JEM, TM, XG, NY, and TN designed and performed experiments, analyzed and interpreted data, and drafted and critically revised the manuscript; MSO contributed to data analysis and interpretation; and all authors gave final approval and agree to be accountable for all aspects of the work.

## Supporting information


**Table S1**. Sex‐enriched protein‐coding genes differentially expressed in murine parotid, sublingual, and submandibular glands.

**Table S2**. Enriched GO terms associated with sex‐wise differentially expressed protein‐coding genes in murine parotid, sublingual, and submandibular salivary glands.

**Table S3**. Co‐expression of differentially expressed SEGs vs. differentially expressed TF genes in the murine major salivary glands.

**Table S4.** Enriched GO terms for differentially expressed protein‐coding genes that correlated with the expressed TFs in the SLG and SMG.

**Table S5**. Gene expression of ion transport‐related proteins in murine parotid, sublingual and submandibular salivary glands.
