# Supplementary material for: Sexual dimorphisms in the transcriptomes of murine salivary glands
Source: FEBS Open Bio. 2019 Mar 30;9(5):947–58. doi: 10.1002/2211-5463.12625 (PMC6487692; doi:10.1002/2211-5463.12625)
Supplement: Supplementary file 5 — Table S5. Gene expression of ion transport‐related proteins in murine parotid, sublingual and submandibular salivary glands. [file FEB4-9-947-s005.pdf]

Supplemental Tables 5A-C. Gene expression of ion transport-related proteins in murine parotid, sublingual and submandibular glands.

The gene names of 25 ion and water transport proteins are listed with the FPKM expression values by gland type **(*5A*)** and by sex **(*5B*)**, where n = 6 for each gland type, parotid (PG), sublingual (SLG) and submandibular (SMG) glands, and n = 3 for each sex. *FPKM in each gland is significantly different from FPKM in the other glands. Fold changes with *q*<0.05 were considered statistically significant. ***5C.*** Quantitative Real-Time qPCR of NaCl reabsorption-related genes expressed in murine parotid, sublingual and submandibular glands was performed to determine the mRNA levels for members of the Na^+ channel *Scnn1* gene family (*a, b* and *g*) and the Cl^- channel *Cftr*. Mean values are shown for glands from 4 male and 4 female mice. The *β-actin* mRNA level was selected as reference for standard -ΔΔCq normalization. Expression correlation across the salivary gland samples was calculated for individual genes between the qPCR abundance values and RNA-seq FPKM values, where r = 0.92, 0.97, 0.99 and 0.97 for *Scnn1a* , *Scnn1b*, *Scnn1g* and *Cftr*, respectively. Unpaired student's *t*-test statistical analysis was used to compare male vs. female data for individual genes where *p<0.01.

Supplemental Table 5A. FPKMs by Gland

Gene ID	PG	SMG	SLG
Ano1	****27.12***	11.88	9.46
Slc12a2	93.64	62.87	43.82
Slc26a6	1.18	4.41	****38.57***
Slc9a1	1.89	****5.42***	2.75
Cftr	0.58	****2.76***	0.56
Lrrc8a	2.85	2.59	2.00
Clcn2	0.85	****2.30***	0.39
Kcnn4	****75.65***	31.25	22.86
Kcnma1	5.18	4.47	****1.21***
Scnn1a	1.15	****2.27***	1.06
Scnn1b	5.40	****36.28***	4.59
Scnn1g	2.09	****9.81***	1.25
Aqp5	87.56	69.60	80.03
Chrm1	0.32	0.34	****1.82***
Chrm3	1.32	0.74	1.14
Adrb1	4.21	5.15	****0.41***
Adrb2	1.12	****3.61***	1.30
Bsnd	1.85	****6.83***	1.20
Lrrc26	174.93	121.86	****202.24***
Slc26a2	****2.48***	4.49	5.03
Slc4a2	1.49	1.32	1.36
Slc4a4	0.62	0.46	0.77
Slc4a7	0.52	0.83	1.04
Slc5a5	0.24	0.31	0.05
Slc5a8	9.45	9.10	****17.32***

Supplemental Table 5B. FPKMs by Gland and Sex

Gene ID	PG M	PG F	SMG M	SMG F	SLG M	SLG F
Ano1	27.66	26.57	12.44	11.32	9.50	9.42
Slc12a2	85.93	101.36	50.53	75.20	40.68	46.96
Slc26a6	1.14	1.22	2.37	***6.44**	39.17	37.96
Slc9a1	1.99	1.79	***8.05**	2.79	3.30	2.20
Cftr	0.50	0.66	***4.36**	1.16	***0.86**	0.26
Lrrc8a	3.20	2.51	2.52	2.66	2.04	1.97
Clcn2	0.89	0.82	***3.59**	1.02	0.49	0.30
Kcnn4	77.58	73.73	24.33	38.18	20.48	25.24
Kcnma1	4.24	6.12	3.47	5.48	1.19	1.23
Scnn1a	0.99	1.32	2.86	1.69	1.22	0.90
Scnn1b	5.27	5.52	***57.96**	14.60	***7.91**	1.27
Scnn1g	1.89	2.28	13.15	6.48	***1.96**	0.55
Aqp5	82.39	92.74	47.28	91.91	69.96	90.09
Chrm1	0.24	0.39	0.33	0.34	1.83	1.80
Chrm3	1.42	1.22	0.68	0.80	1.02	1.26
Adrb1	3.54	4.88	3.41	6.89	0.51	0.30
Adrb2	1.02	1.22	4.21	3.00	1.85	0.74
Bsnd	2.18	1.51	8.50	5.15	***1.82**	0.57
Lrrc26	186.92	162.93	109.07	134.66	206.72	197.75
Slc26a2	3.04	1.93	4.96	4.02	5.18	4.87
Slc4a2	1.49	1.49	1.19	1.45	1.34	1.39
Slc4a4	0.52	0.71	0.62	0.29	0.77	0.78
Slc4a7	0.51	0.54	0.69	0.97	0.99	1.10
Slc5a5	0.23	0.26	0.49	0.13	0.09	0.02
Slc5a8	9.77	9.13	7.40	10.80	16.29	18.35

Supplemental Table 5C. qPCR values of *Cftr* and *Scnn1* by Gland and Sex

Gene ID	PG M	PG F	SMG M	SMG F	SLG M	SLG F
Cftr	0.45	0.18	5.69	2.12	0.29	***0.60**
Scnn1a	3.05	2.34	6.73	5.22	3.14	2.86
Scnn1b	2.10	1.39	***24.26**	10.38	0.53	***0.71**
Scnn1g	0.58	0.39	***6.26**	2.46	0.17	***0.27**
